# Functional Outcomes at 90 Days in Octogenarians Undergoing Thrombectomy for Acute Ischemic Stroke: A Prospective Cohort Study and Meta-Analysis

**DOI:** 10.3389/fneur.2019.00254

**Published:** 2019-03-20

**Authors:** Angelos Sharobeam, Dennis John Cordato, Nathan Manning, Andrew Cheung, Jason Wenderoth, Cecilia Cappelen-Smith

**Affiliations:** ^1^Department of Neurology and Neurophysiology, Liverpool Hospital, Sydney, NSW, Australia; ^2^Ingham Institute for Applied Medical Research, Sydney, NSW, Australia; ^3^South Western Sydney Clinical School, University of New South Wales, Sydney, NSW, Australia; ^4^Department of Interventional Neuroradiology, Liverpool Hospital, Sydney, NSW, Australia; ^5^Prince of Wales Clinical School, University of NSW, Sydney, NSW, Australia; ^6^The Florey Institute of Neuroscience, Melbourne, VIC, Australia

**Keywords:** stroke, elderly, endovascular, thrombectomy, outcome

## Abstract

**Background:** Elderly patients account for 30% of acute ischemic stroke (AIS) but are under-represented in randomized controlled trials of endovascular thrombectomy (EVT). Meta-analysis of “real world” studies evaluating 90-day outcomes in elderly patients ≥80 years have been limited to small numbers undergoing EVT with older generation devices.

**Methods:** A retrospective analysis of 181 prospectively collected patients who received EVT for anterior circulation AIS at an Australian center over 2.5-years. The study aims to determine (i) 90-day functional outcomes (modified Rankin Scale mRS 0–2) in patients ≥80 vs. <80 years, (ii) the interaction of prognostic factors and age and (iii) compare our data to those previously reported using a meta-analysis of outcomes in observational studies using second generation thrombectomy devices.

**Results:** We analyzed 2,387 patients (≥80 years, *n* = 649; <80 years, *n* = 1,738) from 14 studies including our study (≥80 years, *n* = 71; <80 years, *n* = 110). Twenty-eight percent of our and 30% of the meta-analysis elderly cohort achieved good 90-day mRS compared to 55 and 52%, respectively of younger patients (*p* < 0.001). Twenty-seven percent of our and 26% of the meta-analysis elderly cohort died compared to 16% (*p* = 0.07) and 15% (*p* < 0.0001), respectively of younger patients. Baseline NIHSS≥16 correlated with poor prognosis in elderly (OR 16.4; 95% CI 4.49–59.91, *p* < 0.001) and younger (OR 8.73;95% CI 3.35–22.80, *p* < 0.001) patients. Prior rt-PA was associated with favorable outcome in younger (OR 2.90; 95%CI 1.29–6.52, *p* = 0.01) patients only.

**Conclusion:** EVT has less favorable outcomes in elderly patients. However, results are better than outcomes in historical controls not treated with thrombectomy providing further support for EVT in the elderly.

## Introduction

Endovascular thrombectomy (EVT) is an established treatment for acute ischemic stroke (AIS) due to a large vessel occlusion (LVO) involving the anterior cerebral circulation in all patients within 6 h (1–6) and selected patients according to advanced imaging findings up to 24 h from stroke onset (Level 1A evidence) (7–9). Increasing age is an independent risk factor for a worse prognosis after AIS (10). Potential reasons include a higher incidence of comorbidities, shorter time for conversion of ischemic penumbra to core (11) and aging of the vascular anatomy (12).

Although elderly patients account for over 30% of stroke admissions (13), they have been under-represented in randomized controlled (RCTs) trials of EVT. The HERMES meta-analysis of the 5 pivotal EVT trials supported the safety and efficacy of EVT in the elderly but included only 198 (15%) patients aged ≥80 years from a pooled total of 1,278 (6). As the numbers of elderly patients presenting with AIS and LVO suitable for EVT are expected to increase with an aging population (14) the benefit of EVT in the elderly becomes more relevant.

To date, meta-analysis of “real world” observational studies focusing on 90-day outcomes between patients ≥80 and those <80 years has been limited to small numbers undergoing EVT with older generation devices and/or intra-arterial therapies.

The present study is a retrospective analysis of a prospectively collected cohort of patients who received EVT for AIS in the anterior cerebral circulation at an Australian comprehensive stroke center (CSC), over a 2.5-year period. The study aims were to determine (i) the proportion of good functional outcomes at 90-days (defined by a modified Rankin Scale mRS 0–2) in patients ≥80 years compared with patients <80 years (ii) the interaction of prognostic factors and age on 90-day functional outcomes following EVT and (iii) a comparison of our data to those previously reported in the literature using a meta-analysis of clinical outcomes and mortality in observational studies using second generation thrombectomy devices (stent retrievers).

## Materials and Methods

Liverpool Hospital (LH) is a CSC in Sydney, Australia. The study was approved by our institution's ethics review committee.

### Patient Selection

Patients that underwent EVT for AIS between January 2016 and June 2018 were identified from a prospectively collected EVT outcomes LH database. Consecutive patients with premorbid functional independence defined by a baseline mRS 0–2 and LVO in the anterior cerebral circulation (middle cerebral artery M1, M2 branches, internal carotid artery or tandem lesions) confirmed on computed tomography angiography (CTA) were included. Patients with basilar, vertebral, anterior cerebral artery or distal middle cerebral artery branch (M3) occlusions and those with spontaneous or post-thrombolysis with recombinant tissue plasminogen activator (r-tPA) recanalization were excluded. EVT was performed under general anesthesia in all cases using a second generation thrombectomy device [Solitaire (6), Trevo (7), or Catch Mini (3)], aspiration catheter, or both.

### Data Collection

Detailed demographic information was collected including age, gender, stroke risk factors {prior transient ischemic attack [TIA]/stroke, history of atrial fibrillation [AF], current smoking, hypercholesterolemia, hypertension, ischemic heart disease [IHD], diabetes mellitus}, baseline National Institute of Health Stroke Scale (NIHSS), pre-morbid functional status defined by the mRS and results of acute and progress imaging. In addition, the prior use of r-tPA, time of symptom onset (or time last seen well) to groin puncture and recanalization, procedure duration, and modified Thrombolysis In Cerebral Ischemia (mTICI) recanalization grading scores were collected. Successful recanalization was defined as a mTICI score of ≥2b.

Functional outcome at 90-days (mRS) was determined at the patient's 3-month post stroke follow-up appointment or by phone call to those unable to attend follow-up. Symptomatic intracranial hemorrhage (sICH) rates were determined according to the Safe Implementation of Thrombolysis in Stroke Monitoring study (SITS-MOST) with >30% hemorrhagic involvement of the infarcted area on the post-treatment scan and associated neurological deterioration from baseline by an increase in NIHSS by ≥4 or leading to death (15).

### Literature Search for Meta-Analysis

A comprehensive literature search was performed for observational studies to include in a meta-analysis of 90-day functional outcomes and mortality rates in patients receiving EVT for AIS. Databases including Medline, Pubmed, Embase, and the Cochrane Library were interrogated using the following terms: “outcomes,” “stroke,” “mechanical thrombectomy,” “thrombectomy,” “90-days,” “3-months,” “elderly,” “octogenarian,” “clot retrieval,” and “stent retriever” in various combinations with Boolean operators “OR” “AND” (14, 16, 17). The literature search was limited to publications from 2012 in order to include studies using second-generation thrombectomy devices. Two co-authors selected the included studies (AS, AC). Differences were resolved by a third author (DC). Searches were conducted with the assistance of a trained medical librarian.

Study inclusion criteria were (i) English language (ii) patients with AIS (iii) EVT using a second-generation thrombectomy device and/or aspiration catheter (iv) availability of good 90-day functional outcome (mRS) data defined as an mRS 0–2 and mortality rates in the two pre-specified age groups ≥80 years vs. <80 years (v) observational studies. Exclusion criteria included (i) review articles or case reports (ii) incomplete data sets or incomplete distinction between the two age groups (iii) studies including patients treated with older endovascular devices or intra-arterial therapies.

## Statistical Analysis

Statistical analyses were performed using SPSS software (Version 23 for Windows, SPSS, Armonk, NY, IBM Corp, USA). Patients were dichotomized according to age, <80 and ≥80 years. Univariate analysis of baseline characteristics between the two age groups was performed using a Student's *t-*test for continuous variables. Fisher's exact test was performed for binary categorical variables. Non-parametric testing was performed to compare median differences between the two age groups for onset to groin puncture, onset to recanalization and procedure times. Univariate logistic regression analysis was performed for binary categorical variables in which odds ratios were calculated. Multivariate logistic regression analysis was performed for all variables with *p-*values ≤ 0.1 to adjust for potential confounders. *P-*values < 0.05 were deemed statistically significant.

Review Manager (RevMan) version 5.3 (Copenhagen: The Nordic Cochrane Centre, The Cochrane Collaboration, 2014) was used to perform a meta-analysis of observational studies reporting 90-day mRS (0–2 vs. ≥3) and mortality outcomes according to age for which the cumulative incidence (event rate) and 95% confidence intervals (CI) were estimated from each study. Outcomes were presented as odds ratios (OR) with 95% CI and *p-*values. Heterogeneity of treatment effect across studies was evaluated by using the *I*^2^ statistic (random effects model), in which *I*^2^ >50% suggests substantial heterogeneity. Publication bias was determined using Egger's test for funnel plot asymmetry.

## Results

Two hundred and twenty-six consecutive patients with premorbid functional independence underwent EVT at LH during the study period. Of these, 45 were excluded due to M3 or vertebrobasilar occlusion, or spontaneous/post r-tPA recanalization.

One-hundred and eighty-one patients were included in the study. Ninety-day mRS follow-up data was available in all patients. Seventy-one patients were ≥80 and 110 were <80 years. The baseline characteristics are shown in Table 1. Stroke risk factors including AF (*p* < 0.001) and IHD (*p* = 0.02) were more common in the elderly group, whilst smoking was more common in the younger group (*p* < 0.001). The median baseline NIHSS scores were similar (18 ≥ 80 vs. 17 < 80 years).

**Table 1A:** Baseline characteristics of study cohort.

	**Age ≥ 80** **(*n* = 71)** **No. (%)**	**Age < 80** **(*n* = 110)** **No. (%)**	***P-*value for difference**
**AGE**			
Mean ± SD	85 ± 4	64 ± 12	
Range	80–96	29–79	
**GENDER**			
Female	39 (55%)	51 (46%)	0.24
Male	32 (45%)	59 (54%)	0.24
IV r-tPA	24 (34%)	49 (45%)	0.14
**ADMISSION NIHSS**			
Median (IQR)	18 (13–24)	17 (10–22)	
Range	3–42	1–28	
**LESION LOCATION**			
M1	40 (56%)	67 (61%)	0.51
M2	12 (17%)	13 (12%)	0.35
ICA	7 (10%)	18 (16%)	0.25
Tandem	12 (17%)	12 (11%)	0.25
**RISK FACTORS**			
Atrial fibrillation	47 (66%)	43 (39%)	< 0.001
Previous stroke/TIA	20 (28%)	18 (16%)	0.05^*^
Hypertension	54 (76%)	70 (64%)	0.09
Current smoker	4 (6%)	30 (28%)	< 0.001
Hypercholesterolaemia	47 (66%)	61 (55%)	0.14
Diabetes	18 (25%)	24 (22%)	0.64
Ischemic heart disease	26 (37%)	23 (21%)	0.02
**RECANALIZATION TIMES (MINUTES)**			
Onset to groin puncture, median [IQR]	270 [198–422]	274 [210–426]	0.39
Groin puncture to recanalization, median [IQR]	35 [21–50]	31 [22–41]	0.47
Onset to recanalization, median [IQR]	300 [243–487]	302 [243–458]	0.35

Significant p-values < 0.05;

* *borderline significance*.

**Table 1B:** Outcome measures.

	**Age ≥ 80** **(*n* = 71)** **No. (%)**	**Age < 80** **(*n* = 110)** **No. (%)**	
**mRS OUTCOME SCORE (90 DAYS)**			
0–2 (good outcome)	20 (28%)	61 (55%)	OR 0.32 (CI 0.19–0.56), *p* < 0.001
0	4 (6%)	17 (15%)	
1	8 (11%)	23 (21%)	
2	8 (11%)	21 (19%)	
3–6 (poor outcome)	51 (72%)	49 (45%)	
3	10 (14%)	12 (11%)	
4	13 (18%)	13 (12%)	
5	9 (13%)	6 (6%)	
6	19 (27%)	18 (16%)	
Successful recanalization (mTICI 2b/ TICI 3)	68 (96%)	107 (97%)	*p* = 0.72
Symptomatic ICH	3 (4%)	5 (5%)	*p* = 0.75

*mRS, modified Rankin Scale at 90-days; Functional outcome score 0–6: 0, nil deficit; 1, minor deficit; 2, mild deficit; 3, moderate deficit; 4, requires help to walk; 5, bed bound and 6 is death; mTICI, modified Treatment In Cerebral Ischemia; Recanalization classification 0–3: 0, (no flow); 2a < 50% flow in re-opened artery; 2b ≥ 50% flow in re-opened artery and 3 (normal flow). mTICI 2b/3 considered successful recanalization measures*.

Twenty-eight percent of elderly patients had a good 90-day functional outcome, compared with 55% in the younger cohort (*p* < 0.001) (Table 1). Symptomatic intracranial hemorrhage rates were similar (4 vs. 5%, *p* = 0.75). A high proportion of each group achieved successful recanalization (96% ≥ 80 vs. 97% < 80 years, *p* = 0.72). These high rates of successful recanalization may reflect local reporting methods, but as noted were not statistically different between age groups. Mortality at 90-days showed a non-statistically significant trend toward being higher in elderly patients (27 vs. 16%, *p* = 0.07).

Baseline characteristics predictive of a poor 90-day outcome in the both cohorts were an admission NIHSS ≥ 16 (elderly: OR 16.4 95% CI 4.49–59.91, *p* < 0.001; younger: OR 8.73, 95% CI 3.35–22.80, *p* < 0.001). Administration of r-tPA prior to EVT was associated with a favorable outcome in younger (OR 2.90, CI 1.29–6.52, *p* = 0.01) but not elderly patients. There was a trend toward poor outcome in elderly patients with a prior stroke/TIA (OR 2.98, 95% CI 0.99–8.98, *p* = 0.07). No other baseline characteristics correlated with outcome (all *p* > 0.10). On multivariate analysis, admission NIHSS ≥ 16 remained a significant predictor of poor outcome in both age groups (*p* < 0.001). Prior r-tPA remained a significant predictor of a good outcome in the younger cohort (*p* = 0.02) but prior stroke/TIA was not a significant predictor of poor outcome in the elderly (*p* = 0.3).

Logistic regression analysis of the interaction between age (<80 years vs. ≥ 80 years) and NIHSS≥ 16 failed to identify a statistically significant association between the two (*p* = 0.5). NIHSS ≥ 16 (OR 9.7; 95% CI 4.7–20.2, *p* < 0.001) was a stronger predictor of 90-day functional outcome than age ≥80 years (OR 3.8, 95% CI 1.8–8.2, *p* < 0.001) when the interaction of both variables was analyzed.

There was no difference in time from symptom onset to groin puncture (*p* = 0.39), procedure time (*p* = 0.47) or symptom onset to recanalization (*p* = 0.35) between the two groups (Table 1). The most common vessel occlusion site in both cohorts was an M1 occlusion.

## Meta-Analysis

**Figure 1:**
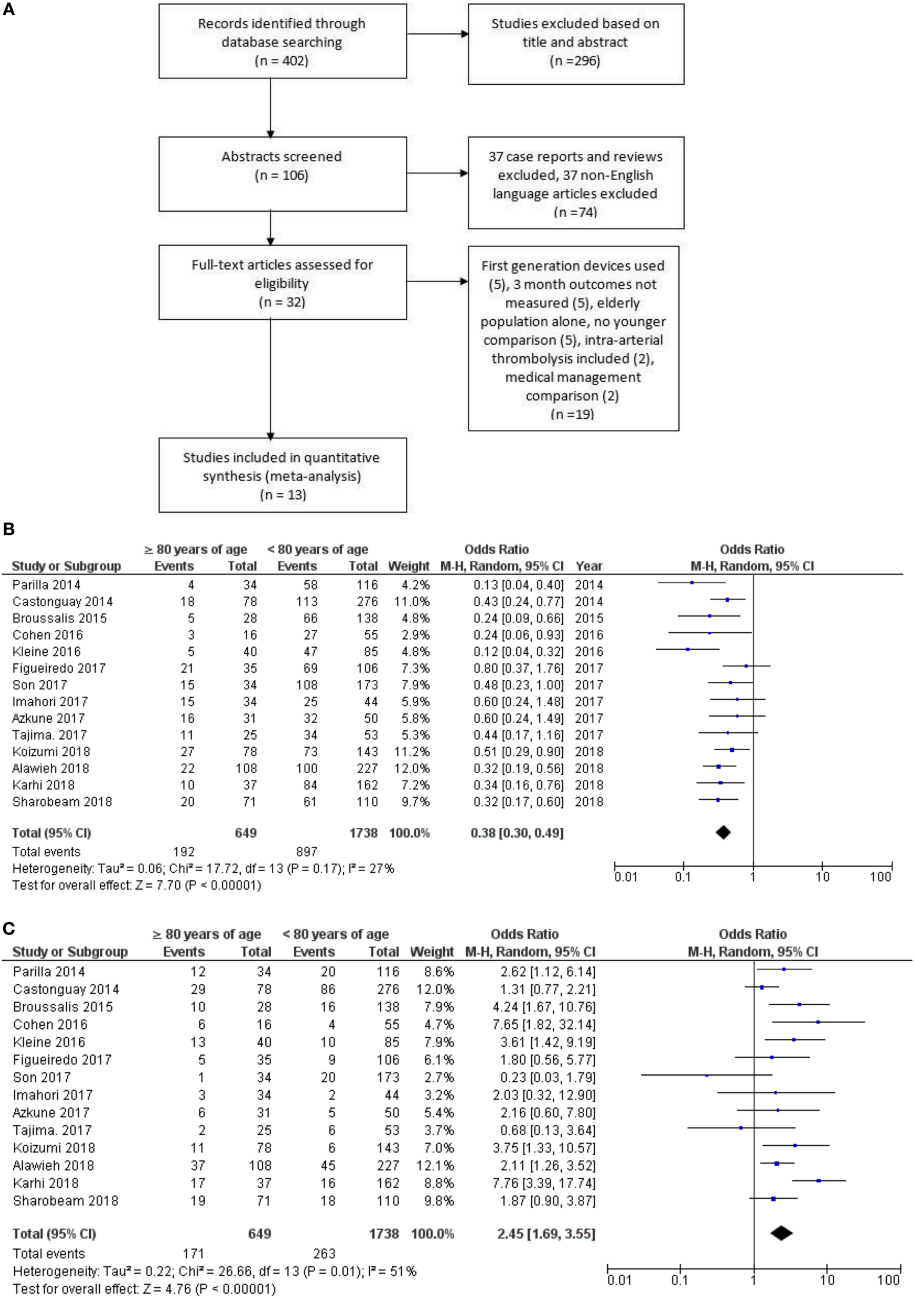
**(A)** Flow chart for study selection into meta-analysis. **(B)** Elderly vs. young pooled study data showing poorer 90 day outcome and **(C)** higher mortality in the elderly group.

A flow diagram of the search process is shown in Figure 1A. The literature search yielded 402 results. After review of abstracts, titles and exclusion of non-English papers, reviews and case reports, 32 full text articles were selected for full text screening, of which 13 studies met the study inclusion criteria (12, 18–29) and were selected for analysis. A total of 2,387 patients (≥80 years, *n* = 649 vs. <80 years, *n* = 1,738) from 14 studies including our study, had data on 90-day good clinical outcomes and mortality after EVT. Good clinical outcome (mRS 0–2) was less common (OR, 0.38; 95% CI: 0.30–0.49; *p* < 0.00001) and mortality higher (OR 2.45; 95% CI: 1.69–3.55; *p* < 0.00001) at 90-days in patients aged ≥80 years (Figures 1B,C).

There was no significant heterogeneity between studies evaluating 90-day clinical outcome (*I*^2^ = 27%, *p* = 0.17). Studies evaluating mortality had evidence of heterogeneity (*I*^2^ = 51%, *p* = 0.01). Egger's test for funnel plot asymmetry did not show evidence of publication bias (90-day good outcomes: intercept = −0.70; *t* = −0.35, *p* = 0.73; and mortality: intercept = −0.18, *t* = −0.11, *p* = 0.91). Funnel plots are provided as Supplementary Material.

Subgroup analysis of studies was performed according to whether data was prospectively collected and found similar outcome and mortality results (see Supplementary Material).

## Discussion

The findings of our study and meta-analysis indicate a lower proportion of elderly patients achieve functional independence following EVT for AIS due to LVO in the anterior circulation. Elderly patients also carry a 2-fold increased risk of death. Despite these findings, 28% of our elderly cohort achieved a good 90-day functional outcome. In our study, successful recanalization rates were similar and sICH rates were low irrespective of patient age. The single prognostic factor associated with a poor 90-day outcome was a baseline NIHSS ≥ 16 although prior history of stroke/TIA in our elderly cohort showed a trend to worse outcome.

Advanced age (≥80 years) is a predictor of dependency and mortality in AIS due to LVO (30, 31). The natural history of AIS in elderly patients ≥80 years with LVO in the anterior circulation can be extrapolated from non-randomized patients (controls) in RCTs. In the HERMES collaboration, favorable outcome was 13.9% and mortality 45% for patients aged ≥80 years randomized not to receive EVT (6). Our meta-analysis is the largest pooled analysis of 90-day outcomes in elderly patients undergoing EVT with second generation thrombectomy devices and supports the findings of RCTs that the rates of favorable outcome (30% in our meta-analysis and 29.8% in HERMES) is superior and mortality (26% meta-analysis and 28% HERMES) less than elderly AIS patients randomized not to receive EVT in RCTs.

Baseline stroke severity was associated with worse outcome and a stronger predictor of 90-day functional independence in elderly compared to younger patients. Possible explanations for the relatively poorer age-related functional outcome found in our study and on meta-analysis include a higher incidence of comorbidities (22) such as AF, prior stroke/TIA and IHD as in our study. Other age-related factors may include reduced neurological reserve and/or neuroplasticity (32), less penumbra (11) and a lower rate of r-tPA administration (33, 34). In addition, although we found no age-related difference in procedural times in our cohort, other studies have reported a higher likelihood of arterial tortuosity which may prolong the time to successful recanalization (12).

The limitations of our study include its retrospective design and relative small patient numbers. In addition certain variables which may influence outcome including stroke sub-type, lesion volume and collaterals were not examined. The authors also acknowledge that other less restrictive definitions of sICH were not used in this study (35). The limitations of the meta-analysis included the possibility of publication bias with more experienced centers (with better results) more likely to publish findings and the retrospective nature of included studies which carries a risk of selection bias. In addition, it was not possible to stratify outcomes according to premorbid mRS exclusion range (not defined in 6 of the 13 studies), comorbidities, advanced imaging findings, type of anesthesia (not defined in 2 studies) and stroke onset to groin puncture times. Despite these limitations, the meta-analysis included 649 elderly patients treated with second generation thrombectomy devices and there was no significant heterogeneity for 90-day outcomes.

## Conclusion

Our study and the meta-analysis show improved functional outcomes and reduced mortality rates compared to control elderly patients (randomized not to receive EVT) in RCTs (6). These findings may assist informed consent regarding the likelihood of functional independence with EVT in the elderly. Further research is needed to determine the cost-benefit and refine selection criteria in this age group.

## Data Availability

The datasets generated for this study are available on request to the corresponding author.

## Author Contributions

AS was responsible for the primary writing of the text, performed the data collection, interpretation, and analysis. DC and CC-S were responsible for the study design, ethics approval and were involved in writing, editing, and critical appraisal of the text. NM, AC, and JW performed all the procedures reported in this study and were involved in editing and critical appraisal of the text.

### Conflict of Interest Statement

AC, NM, and JW are consultants for Medtronic Neurovascular, Stryker Neurovascular and Microvention. The consulting positions played no role in the study design, the collection, analysis, or interpretation of data, the writing of this paper or the decision to submit it for publication. The remaining authors declare that the research was conducted in the absence of any commercial or financial relationships that could be construed as a potential conflict of interest.
