# Supplementary material for: Functional Outcomes at 90 Days in Octogenarians Undergoing Thrombectomy for Acute Ischemic Stroke: A Prospective Cohort Study and Meta-Analysis
Source: Front Neurol. 2019 Mar 20;10:254. doi: 10.3389/fneur.2019.00254 (PMC6435519; doi:10.3389/fneur.2019.00254)
Supplement: Supplementary file 1 [file Data_Sheet_1.docx]

**Supplementary 1- Meta-analysis of pooled studies- funnel plots**


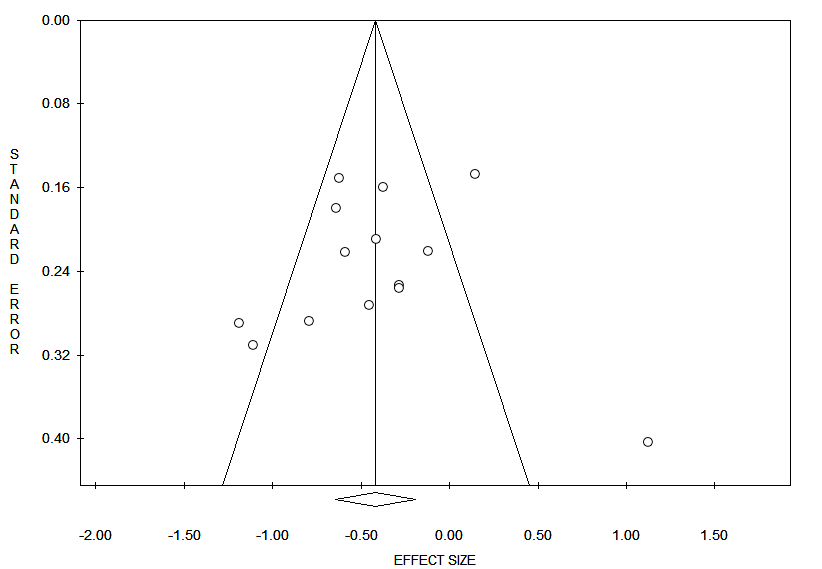


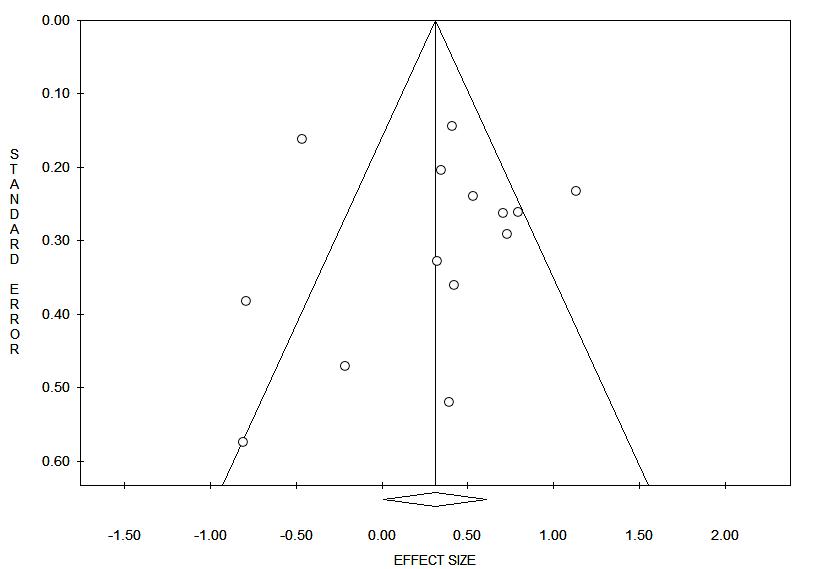


**Figure S1(A)** 90-day good functional outcome funnel plot for all studies.

There was no evidence of publication bias with respect to 90-day good functional outcome

(Intercept = -0.70, t= -0.35, p=0.73) **(B)** 90-day mortality funnel plot for all studies.

There was no evidence of publication bias with respect to 90-day mortality (Intercept = -0.18, t= -0.11, p=0.91).

**Supplementary 2- Meta-analysis of prospective studies- Forest plots**


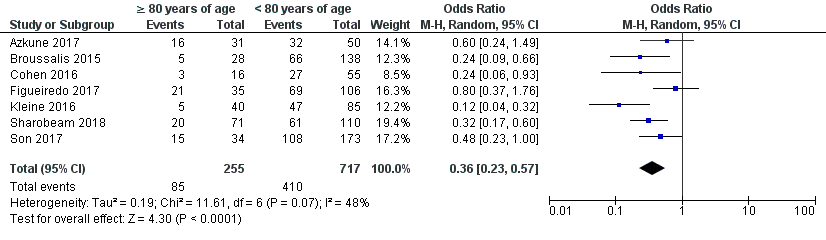


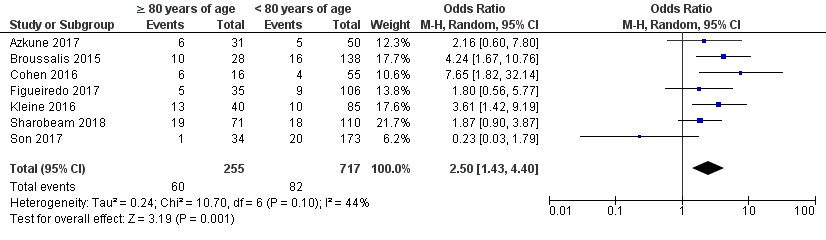


**Figure S2(A)** 90-day good functional outcome forest plot of prospective studies. There is no significant heterogeneity between studies (OR 0.36 [0.23-0.57], I^2^=48%, p=0.07).

**(B)** 90-day mortality forest plot of prospective studies. There is no significant heterogeneity between studies (OR= 2.50[1.43- 4.40], I^2^=44%, p=0.10).

**Supplementary 3- Meta-analysis of prospective studies- funnel plots**

**
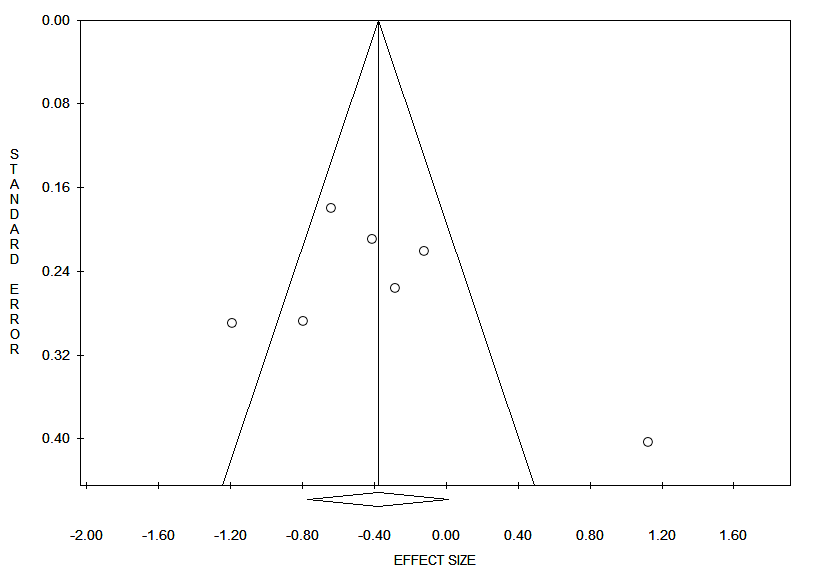
**

**
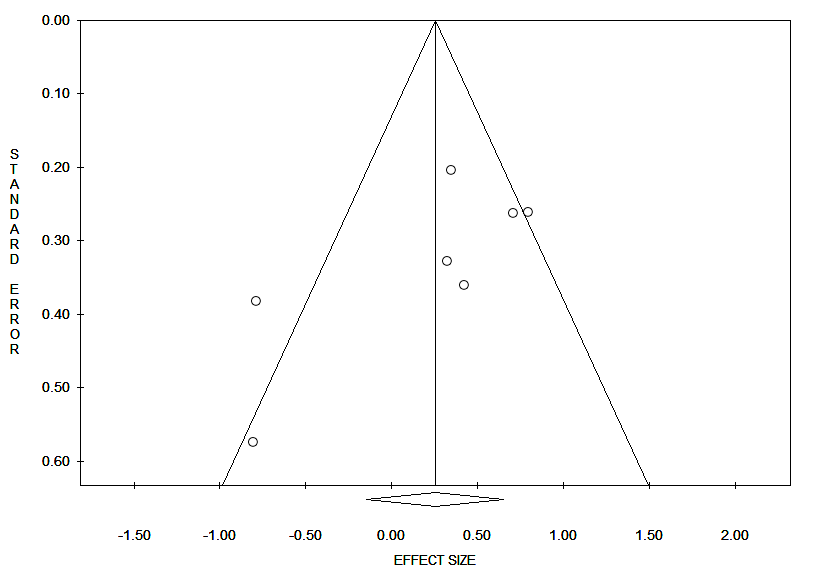
**

**Figure S3(A)** 90-day good functional outcome funnel plot for prospective studies.

There was no evidence of publication bias with respect to 90-day good functional outcome (Intercept= 3.24, t= 0.91, p=0.40) **(B)** 90-day mortality funnel plot for prospective studies. There was no evidence of publication bias with respect to 90-day mortality (Intercept= -3.64, t= -1.87, p=0.12).

**Supplementary 4- Meta-analysis of retrospective studies- forest plots**


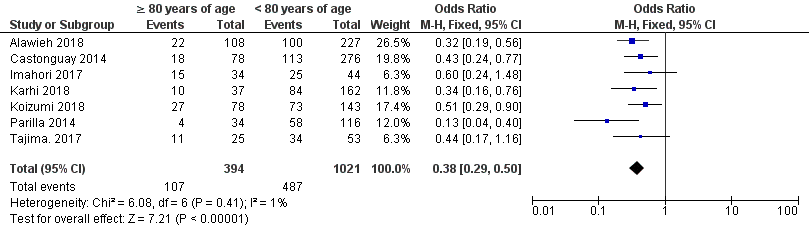


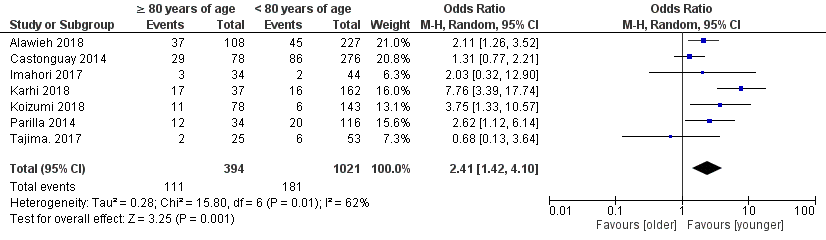


**Table S4 (A)** 90-day good functional outcome forest plot of retrospective studies. There is no significant heterogeneity between studies (OR= 2.41[1.42-4.10], I^2^=1%, p=0.41).  **(B)** 90-day mortality forest plot of retrospective studies. There is significant heterogeneity between studies (OR= 0.38[0.29-0.50], I^2^=62%, p=0.01).

**Supplementary 5- Meta-analysis of retrospective studies- funnel plots**

**
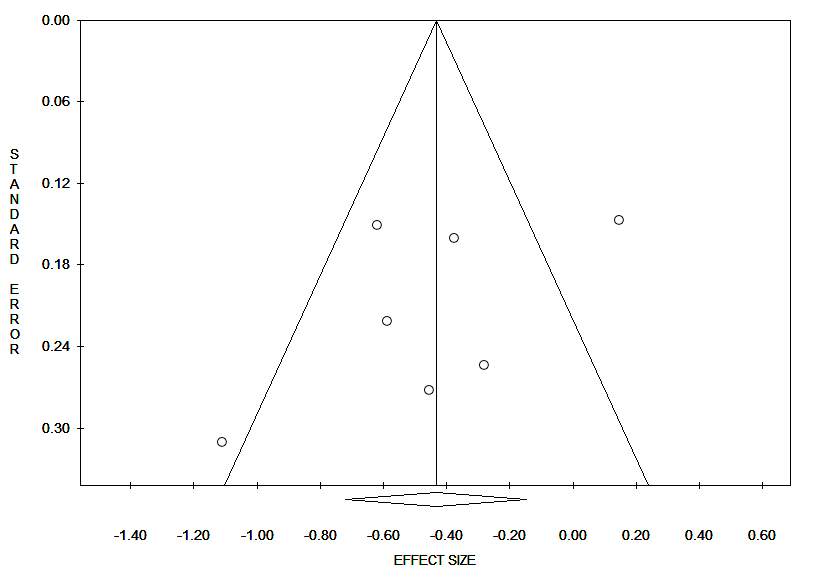
**


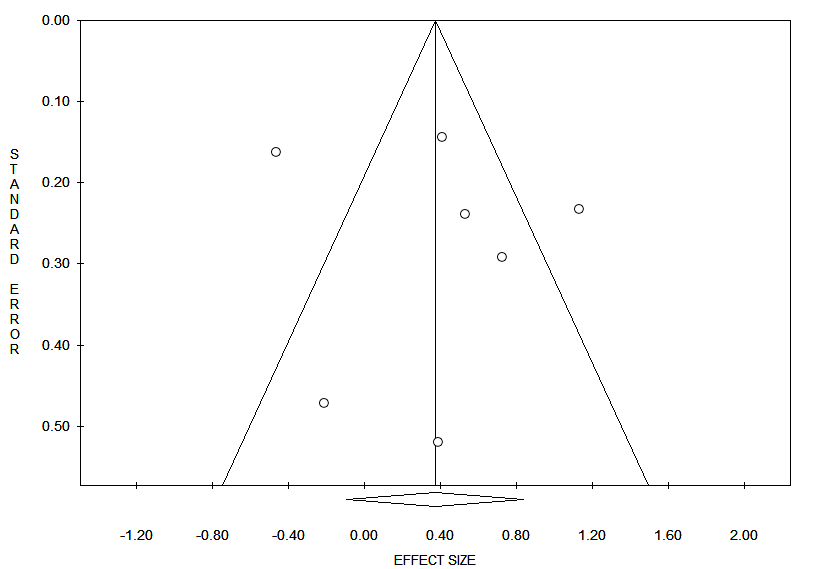


**Figure S5**(**A**) 90-day good functional outcome funnel plot of retrospective studies.

There was no evidence of publication bias with respect to 90-day good functional outcome (Intercept= -3.24, t= -1.27, p=0.26). (**B**) 90-day mortality funnel plot of retrospective studies. There was no evidence of publication bias with respect to 90-day mortality (Intercept= 1.32, t= 0.49, p=0.64).
